# Meeting of United States Veterinary Association

**Published:** 1890-02

**Authors:** W. Horace Hoskins

**Affiliations:** Secretary; 12 S. Thirty-seventh Street, Phila.


UNITED STATES VETERINARY MEDICAL ASSOCIATION,
PHILADELPHIA, JAN. 20th, 1890.

Editors J. C. M. and V. A.,

*Dear Sirs:*—I beg leave to announce that the Comitio Minora will meet in session in February to determine the place of meeting for the annual gathering of the United States Veterinary Medical Association.

Those desirous of offering inducements or pleas for the place of meeting, can, by communicating with the Secretary, have a hearing before the committee, or any proposition submitted, entrusted to his care.

W. HORACE HOSKINS, *Secretary*,
12 S. Thirty-seventh Street, Phila.

